# Looking Beyond Our Similarities: How Perceived (In)Visible Dissimilarity Relates to Feelings of Inclusion at Work

**DOI:** 10.3389/fpsyg.2019.00575

**Published:** 2019-03-27

**Authors:** Onur Şahin, Jojanneke van der Toorn, Wiebren S. Jansen, Edwin J. Boezeman, Naomi Ellemers

**Affiliations:** ^1^Department of Social, Health and Organizational Psychology, Utrecht University, Utrecht, Netherlands; ^2^Department of Social and Organizational Psychology, Institute of Psychology, Leiden University, Leiden, Netherlands

**Keywords:** dissimilarity, inclusion, climate for inclusion, surface-level, deep-level

## Abstract

We investigated how the perception of being dissimilar to others at work relates to employees’ felt inclusion, distinguishing between surface-level and deep-level dissimilarity. In addition, we tested the indirect relationships between surface-level and deep-level dissimilarity and work-related outcomes, through social inclusion. Furthermore, we tested the moderating role of a climate for inclusion in the relationship between perceived dissimilarity and felt inclusion. We analyzed survey data from 887 employees of a public service organization. An ANOVA showed that felt inclusion was lower for individuals who perceived themselves as deep-level dissimilar compared to individuals who perceived themselves as similar, while felt inclusion did not differ among individuals who perceived themselves as surface-level similar or dissimilar. Furthermore, a moderated mediation analysis showed a negative conditional indirect relationship between deep-level dissimilarity and work-related outcomes through felt inclusion. Interestingly, while the moderation showed that a positive climate for inclusion buffered the negative relationship between deep-level dissimilarity and felt inclusion, it also positively related to feelings of inclusion among all employees, regardless of their perceived (dis)similarity. This research significantly improves our understanding of how perceived dissimilarity affects employees by distinguishing between surface-level and deep-level dissimilarity and by demonstrating the importance of a climate for inclusion.

## Introduction

The sharp increase in workforce diversity during the last decades presents important challenges for organizations and employees to overcome. A well-established finding is that dissimilarity between individuals can impede mutual trust and understanding, and challenge social integration in the workplace, which have been associated with (team) performance losses and increased employee turnover (Chattopadhyay, 1999; Garrison and Wakefield, 2010; Guillaume et al., 2012). Dissimilarity between workers has been related to surface-level (relatively visible or readily detected) attributes such as gender, age, and ethnicity, or to deep-level (less visible or underlying) attributes such as beliefs and values (Phillips and Loyd, 2006; Jackson and Joshi, 2011; Guillaume et al., 2012; Mor Barak et al., 2016). In the current research, we will not examine the objective classification of specific attributes. Instead, we will address employees’ subjective perceptions of their surface-level and deep-level dissimilarity to other people at work. We will also not focus on a specific comparison group (e.g., a specific target group such as direct colleagues, supervisors or customers), but rather are interested in employees’ general perception of being dissimilar to most others at work.

Even though prior work suggests that surface-level and deep-level dissimilarity are both negatively related to work outcomes, the ways in which they impact employees are likely to differ. For example, surface-level dissimilarity has been shown to have a negative effect on social integration only under low team interdependence, while deep-level dissimilarity had a stronger negative effect on social integration under high interdependence than under low interdependence (Guillaume et al., 2012). This suggests that the two types of dissimilarity can have different effects, and/or that their effects depend on different moderating factors. Yet, the correlates and implications of these different types of dissimilarity have not been systematically established. Hence, we do not yet know whether surface-level or deep-level dissimilarity is more predictive of employees’ sense of inclusion and its downstream work-related consequences. It is also unknown whether they operate independently, buffer, or reinforce one another. Furthermore, while previous research has indicated that an inclusive work climate buffers the negative effects of surface-level dissimilarity on inclusion (Jansen et al., 2017), it is unclear whether the negative effects of deep-level dissimilarity can be mitigated in similar ways. Answering these questions is highly important considering that employees likely differ from others at work in terms of both surface-level and deep-level dimensions. Hence, this study contributes to existing knowledge by investigating the separate and joint influences of surface-level and deep-level dissimilarity on social inclusion, as well as the moderating role of the work climate in these relationships.

### Dissimilarity at Work

As indicated above, dissimilarity has been found to negatively affect a variety of work outcomes (Hobman et al., 2004; Liao et al., 2008; Guillaume et al., 2012). Hobman et al. (2004), for example, found that employees who perceived themselves to have a different demographic profile than their colleagues (i.e., in terms of visible and informational characteristics) were less involved in their workgroup. Liao et al. (2008), furthermore, found perceived deep-level dissimilarity on the basis of personality to be associated with worse job attitudes, less helping behavior, greater work withdrawal, and greater voluntary turnover.

There are several mechanisms through which dissimilarity is thought to affect employees. One mechanism concerns ingroup bias on the part of numerical majority members, leading them to discriminate against and otherwise mistreat those who are dissimilar to them (Van Laer and Janssens, 2011; Williams and Dempsey, 2014; Drydakis, 2015; Waldring et al., 2015; Midtbøen, 2016; Mishel, 2016; Van den Berg et al., 2017; Yavorsky, 2017). Another mechanism, observed among numerical minority members, relates to their increased monitoring of the self and the environment; Employees representing a numerical minority tend to be more engaged in monitoring their performance and the workplace for cues about who belongs and who does not. Their preoccupation with social acceptance cues diverts cognitive resources away from task performance and has important work-related consequences (Murphy et al., 2007; Guillaume et al., 2014; Master et al., 2016; see also Ståhl et al., 2012). Even cues that are not intended to exclude people, such as all-White conference speakers or pictures of male leaders in the company canteen, might undermine performance and lower feelings of inclusion among those not represented by these cues (Latu et al., 2013; Cheryan et al., 2014; Murphy et al., 2018). Furthermore, through the mechanism of similarity-attraction (Byrne, 1997), minority members may self-segregate into minority subgroups. This process is stronger in people who are more aware of their minority status (Schmader and Sedikides, 2018) and, by further detaching them from others at work, adds to the disadvantages that dissimilar people face through the mechanisms discussed above.

Of the previous work studying the relationship between dissimilarity and work outcomes, some studies used objective measures of dissimilarity (e.g., quantifying the degree of dissimilarity based on the demographic composition of work teams, Jansen et al., 2017) while others used subjective measures (e.g., asking participants whether they feel dissimilar to other team members; Hobman et al., 2004). Because we are interested in the experiences of employees, and because several studies indicated perceived dissimilarity to have stronger effects than actual dissimilarity (Turban et al., 1988; Strauss et al., 2001), the current research utilizes a subjective measure of dissimilarity.

In the current study, we use the terms “surface-level” and “deep-level” to capture the full range of attributes that could lead to perceived dissimilarity in the work context, because these were used to study dissimilarity in previous research (e.g., Guillaume et al., 2012). These attributes can include age, ethnicity, gender, beliefs, values, or sexual orientation. We acknowledge it is not self-evident whether an attribute is surface-level or deep-level, or both. This can depend on many factors, such as the extent to which the attributes are expressed in overt behavior or verbally acknowledged. Furthermore, the degree to which people perceive themselves to be surface-level and/or deep-level dissimilar to others can be indicated by multiple attributes they have as well as the intersection of these attributes. For example, employees who are bisexual could perceive themselves as surface-level and/or deep-level dissimilar to their heterosexual colleagues, which may, for example, depend on whether they have a same-sex or opposite-sex partner. Transgender employees might perceive themselves to be deep-level dissimilar in terms of their gender identity, while their perception of surface-level dissimilarity may depend on the particulars of their gender expression. Both surface-level and deep-level dissimilarity were shown to have a negative relationship with important work-related outcomes, such as employee performance and turnover (Guillaume et al., 2012), work group involvement (Hobman et al., 2004) and helping behavior (Liang et al., 2015).

Even though the relationship between dissimilarity and work-related outcomes is widely studied, very little research has focused on the effects of dissimilarity on employees’ sense of social inclusion at work. The construct of social inclusion refers to individuals’ perception that they belong and can be their authentic selves in a particular context (Jansen et al., 2014), such as the workplace. Understanding the relationship between dissimilarity and inclusion at work is important, since inclusion has been related to several outcomes that may have far-reaching implications for both employees and organizations, such as well-being and performance (Sønderlund et al., 2017; Chen and Tang, 2018). One study that did examine the relationship between gender dissimilarity and felt inclusion is the research by Jansen et al. (2017), which demonstrated a lower sense of belonging and authenticity among those who diverged more (versus less) from the rest of the work team in terms of gender. This prior work is limited, however, in the sense that it addressed actual dissimilarity rather than subjectively perceived differences, and only focused on a single surface-level characteristic, namely gender. With the current research, we aim to contribute to the organizational diversity literature by examining the separate and interactive effects of perceptions of surface-level and deep-level dissimilarity on employees’ feelings of inclusion. Because previous research demonstrated felt social inclusion to relate to important work outcomes (e.g., Derks et al., 2007; Jansen et al., 2017; Chen and Tang, 2018), we will not only address social inclusion, but additionally investigate its relationships with job satisfaction, work-related stress, turnover intentions, career commitment and career advancement motivation in the organization.

Whether surface-level and deep-level dissimilarity differentially affect employees and whether they reinforce one another is not only of theoretical importance but also of practical relevance because surface-level and deep-level dissimilarity are not necessarily overlapping or independent. Employees may both look different than others at work (e.g., in terms of skin color suggesting a different ethnicity) and hold different values to them, but it is also possible that they look very similar yet hold different values or that they look very different yet hold the same values. Hence, it is important to disentangle their separate and joint effects.

Based on the research summarized above, we anticipate that – in principle – both types of perceived dissimilarity will be negatively related to feelings of inclusion. As no previous work has addressed the separate and combined effects of surface-level and deep-level dissimilarity on social inclusion or examined possible differences in their predictive strength, we have no specific hypotheses regarding their relative and interactive effects. These will be investigated in an exploratory fashion.

Feeling included is theorized to satisfy two fundamental human needs, the need to belong and the need to be authentic. Accordingly, inclusion has been found to be vital for employee motivation, performance, and wellbeing (Jansen et al., 2014). More specifically, inclusion was shown to be a key predictor of work satisfaction. This may not be surprising, given that inclusion at work also implies, for example, taking part in informal events or being part of information networks (Waters and Bortree, 2012). Conversely, when employees feel excluded at work, negative effects are likely to occur. Exclusion may increase stress levels (Ryan et al., 2005; Beekman et al., 2016), and is arguably a reason for employees to leave the organization. That is, employees whose fundamental inclusion needs are frustrated may be less likely to stay in their current situation. Preliminary evidence of this relationship comes from research showing that dissimilarity positively relates to turnover intentions, but this relationship is weaker if the organizational climate is supportive of diversity (Gonzalez and Denisi, 2009), likely because such a climate facilitates a sense of inclusion. For these reasons, we hypothesize that feelings of inclusion will mediate the relationship between perceived dissimilarity on the one hand and job satisfaction, work-related stress and turnover intentions on the other.

Recent qualitative research on the career ambitions of women in traditionally masculine environments (i.e., making it likely that they feel dissimilar to their colleagues at work) indicated that women who reported decreased belonging and authenticity, indicating a lack of perceived inclusion, also expressed little ambition to move up the organizational ladder (Sealy and Harman, 2017). Furthermore, stigmatized groups who do feel devalued at work were found to have lowered motivation to perform and grow in the organization (Derks et al., 2007). To further explore the relationship between inclusion and career ambition, we also included the career advancement motivation in the organization as a relevant work outcome in our research. In addition, we address the implications of perceived dissimilarity and felt inclusion for the degree to which participants are committed to their career. This is based on recent findings indicating a link between inclusion and organizational commitment (Harrison et al., 1998; Chen and Tang, 2018).

In summary, we derive the following hypotheses:

H1a: Perceived surface-level and deep-level dissimilarity negatively relate to felt inclusion.
H1b: Perceived surface-level and deep-level dissimilarity negatively relate to key work-related outcomes, namely job satisfaction, work-related stress, turnover intentions, career commitment, and career advancement motivation.
H2: Felt inclusion mediates the relationships between perceived dissimilarity and work-related outcomes.

### Climate for Inclusion

Even though a gloomy picture indicating the negative effects of dissimilarity emerges from prior research, there are also studies suggesting that dissimilarity is not necessarily detrimental to employees. Some previous work has indicated that diverse teams enjoy more beneficial work outcomes when they perceive their organizational climate as inclusive (Nishii, 2013; Bodla et al., 2016; Li et al., 2017). An inclusive climate ensures fair and unbiased treatment of employees, is open toward and values differences between employees, and includes all employees in decision making (Nishii, 2013). There is some indication that the benefits of such an organizational climate may also apply to feelings of social inclusion. Jansen et al. (2017) found that perceiving the work environment to be open toward and appreciative of differences (i.e., as a “diversity climate”) was positively associated with felt inclusion for all employees, but more strongly so for those who were highly dissimilar to most others. In fact, perceiving a positive diversity climate buffered the negative effect of gender dissimilarity on feelings of inclusion, such that dissimilarity was only related to reduced inclusion when employees perceived a negative diversity climate. These findings can likely be generalized to a climate for inclusion since the latter subsumes the diversity climate notion of openness toward and appreciation of differences. Accordingly, we expect that a positive climate for inclusion will, similarly, shield employees from the negative effects of perceived dissimilarity on inclusion.

H3a: Perceived climate for inclusion moderates the relationship between perceived dissimilarity and felt inclusion, such that the negative relationship between perceived dissimilarity and felt inclusion is weaker the more inclusive the climate is perceived to be.
H3b: Perceived climate for inclusion positively relates to felt inclusion.

## Materials and Methods

### Participants

This study was carried out in accordance with the recommendations of the Psychology Research Ethics Committee (PREC) at Leiden University. All participants gave informed consent in accordance with the Declaration of Helsinki. The protocol was approved by the PREC. All employees of a governmental organization in the Netherlands, approximately 4000 people, were invited to participate in our online study. Of these people, 1326 employees opened and started the questionnaire. Our study sample consisted of the 887 employees who completed the questionnaire (40.34% male, 58.53% female, 1.13% chose not to answer this question, 0.23% missing, *M*_age_ = 45.61, *SD*_age_ = 11.80). Participants had been working at the organization for 12.47 years on average (*SD* = 10.55) and worked 32.50 hours a week on average (*SD* = 5.02). Furthermore, 10.50% of participants held a senior position (0.11% missing), 4.63% were trainees (2.59% missing), and 82.64% neither held a senior position nor was a trainee. The sample was relatively highly educated, with 41.66% having completed university education, 37.74% having completed higher professional education, 16.57% having completed middle vocational education, 1.27% having completed lower vocational education and 2.76% having completed secondary education (2.03% missing).

### Procedure and Measures

The organization’s employees received an email with a link to our on-line survey. After providing informed consent, participants first completed a demographics form, which asked them to indicate their sex, age, educational level, tenure, number of hours work per week and whether they are a senior or trainee. These questions were followed by measures of perceived dissimilarity, perceived climate for inclusion, felt inclusion, job satisfaction, work-related stress, turnover intention, career commitment, and career advancement motivation.^1^

#### Perceived Dissimilarity

Perceived dissimilarity was measured using two items, which were adapted from the work of Hobman et al. (2004). To assess surface-level dissimilarity, participants were asked whether they perceived themselves to be *visibly* dissimilar to others at work: “In terms of visible characteristics (e.g., age, sex, ethnicity), I am different than most others at work.” To assess perceived deep-level diversity, they were asked whether they perceived themselves to be *invisibly* different to others at work: “In terms of invisible characteristics (e.g., beliefs, preferences), I am different than most others at work.” The answer options provided were “*yes*” and “*no*,” resulting in the possibilities of being dissimilar in both deep-level and surface-level terms, being dissimilar in either deep-level or surface-level terms and lastly being similar to most others.^2^

#### Perceived Climate for Inclusion

The extent to which participants perceived the climate to be inclusive was measured using a 12-item scale that was developed to capture how people think about, talk about and treat others who are dissimilar to most others. This questionnaire was developed as a screener of climate for inclusion. Participants were asked to indicate how “people who are visibly or invisibly dissimilar than most others” are being treated at work. They did so on a bipolar scale by indicating the extent to which they agreed more with the statement on the left side or with the statement on the right side. The scores ranged from 1 (agreeing most with the left statement) to 7 (agreeing most with the right statement) with a higher score indicating a more inclusive climate. Examples of items are: “They are being disadvantaged at work when making decisions about tasks, salary, etc. – They are being taken into account when making decisions about tasks, salary, etc.,” “They are being seen as an inconvenience – They are being seen as an asset,” and “They are being treated worse than others – They are being treated as people that are valuable” (α = 0.96).

#### Felt Inclusion

The extent to which participants felt included at work was measured with the Perceived Group Inclusion Scale (PGIS; Jansen et al., 2014). This 16-item scale consists of two subscales (belonging and authenticity), which in turn each comprised two components. Belonging comprised group membership (e.g., “People at work give me the feeling that I am part of this group.”) and group affection (e.g., “People at work like me”). Authenticity comprised room for authenticity (e.g., “People at work allow me to be who I am.”) and value in authenticity (e.g., “People at work encourage me to be who I am.”). Each component consists of four items. An exploratory factor analysis (EFA) with oblique (Oblimin) rotation indicated that all items loaded highly on a single factor with all factor loadings exceeding 0.80 (see Supplementary Table A for factor loadings of the one-factor solution). In line with the theoretical components, the parallel analysis (PA) confirmed that four factors with significant Eigenvalues could be distinguished (see Supplementary Table B for the factor loadings on four factors). In the current study, we used inclusion as a single variable because the four factors (group membership, group affection, room for authenticity, and value in authenticity) are the theoretical subdimensions of inclusion (Jansen et al., 2014). The response options ranged from 1 (*completely disagree*) to 7 (*completely agree*) with a higher score indicating that participants felt more included (α = 0.97).

#### Job Satisfaction

The extent to which participants were satisfied with their job was assessed with the three items used by Mitchell et al. (2001): “All in all, I am satisfied with my job,” “In general, I enjoy my job” and “I am very satisfied with my job.” The last item was slightly adapted, as it originally referred to workplace satisfaction instead of job satisfaction. The response options ranged from 1 (*completely disagree*) to 7 (*completely agree*). A higher score indicated more job satisfaction (α = 0.92).

#### Work-Related Stress

We measured participants’ work-related stress with a scale developed by Hadzibajramovic et al. (2015). Participants indicated how they felt at the end of a work day, using the following six items: “calm,” “rested,” “relaxed,” “tense,” “stressed,” and “pressured.” The response options ranged from 1 (*not at all*) to 6 (*very much*). The last three items were reverse-coded, such that a lower score on the scale indicated more stress (α = 0.92).

#### Turnover Intentions

The turnover intentions of participants were measured with a scale developed by Van Velthoven and Meijman (1994), consisting of four questions that the participants could answer with “yes” or “no.” Example items are: “I am planning to change jobs in the coming year,” and “I sometimes think about looking for a job outside this organization.” The answers were coded 0 (*yes*) or 1 (*no*) and the mean score of the four items was taken as the dependent variable. A lower score corresponded to a higher intention to leave (α = 0.76).

#### Career Commitment

The degree to which participants were committed to their career was assessed with a modified version of a scale developed by Ellemers et al. (1998). The scale consisted of six statements, with scores ranging from 1 (*not at all*) to 7 (*very much*). Example items are: “My career plays a central role in my life” and “I think I should have a successful career.” A higher score corresponded to a stronger commitment to one’s career (α = 0.86).

#### Career Advancement Motivation Within Organization

We measured participants’ career advancement motivation using a self-developed scale consisting of five statements, with scores ranging from 1 *(not at all*) to 6 (*very much*). This measure records the willingness of employees to invest in the career at, and on behalf of, the organization. The items are: “I am motivated to exploit all the career opportunities that I will get at this organization,” “I am willing to invest effort to further my development in this organization,” “I am willing to do my best to advance my career in this organization,” “I would like to continue my career in this organization,” and “It is my wish to develop my career in this organization.” A higher score corresponded to a greater career advancement motivation (α = 0.87).

## Results

Analyses were conducted using R software 3.5.1 (R Core Team, 2018), using the *Hmisc* (*v4.1-1;* Harrell, 2018), *car* (*v3.0-2;* Fox and Weisberg, 2011), *sjstats* (*v0,17.0;* Lüdecke, 2018), and *lavaan* (*v0.6-3;* Rosseel, 2012) packages. The full code is available at https://osf.io/exrwd/. The descriptive statistics and zero-order correlations for all variables are displayed in Table 1. A total of 551 (62.12%) participants indicated that they perceived themselves to be similar to their colleagues, 111 (12.51%) perceived themselves as only surface-level dissimilar, 147 (16.57%) perceived themselves as only deep-level dissimilar and 67 (7.55%) perceived themselves as both surface-level and deep-level dissimilar (1.24% missing).

**Table 1:** Descriptive Statistics and Intercorrelations.

	*M*	*SD*	1	2	3	4	5	6	7		8
1. Surface-level dissimilarity	0.20	0.40	–								
2. Deep-level dissimilarity	0.24	0.43	0.16^∗∗∗^	–							
3. Perceived climate for inclusion	4.49	1.00	-0.10^∗∗^	-0.23^∗∗∗^	-						
4. Felt inclusion	5.26	1.09	-0.08^∗^	-0.25^∗∗∗^	0.56^∗∗∗^	–					
5. Job satisfaction	5.67	1.06	0.00	-0.16^∗∗∗^	0.32^∗∗∗^	0.55^∗∗∗^	–				
6. Work-related stress	4.01	0.94	-0.04	-0.13^∗∗∗^	0.26^∗∗∗^	0.37^∗∗∗^	0.40^∗∗∗^	–			
7. Turnover intention	0.64	0.34	0.00	-0.11^∗∗^	0.12^∗∗∗^	0.22^∗∗∗^	0.43^∗∗∗^	0.28^∗∗∗^	–		
8. Career commitment	4.85	1.07	0.10^∗∗^	-0.04	0.10^∗∗^	0.11^∗∗^	0.16^∗∗∗^	-0.04	-0.05	–	
9. Career advancement motivation	4.46	0.87	0.08^∗^	-0.03	0.19^∗∗∗^	0.33^∗∗∗^	0.37^∗∗∗^	0.12^∗∗∗^	0.12^∗∗∗^	0.58^∗∗∗^	


*Dissimilarity was coded as 0 and 1, meaning that the mean scores reflect the percentage of people that perceived themselves as such. ^∗^*p* < 0.05, ^∗∗^*p* < 0.01, ^∗∗∗^*p* < 0.001.*

### Preliminary Analyses

Mardia’s test showed that the assumption of multivariate normality was violated. As a consequence, we used robust test statistics in our CFA and SEM analyses.

To assess whether our measures could be distinguished statistically, we conducted a series of factor analyses.^3^ First, we performed a PA, which yielded nine significant factors. Subsequently, we entered all our Likert-scale measures in an EFA in which we constrained the number of extracted factors to nine (based on the aforementioned PA) and used principal axis factoring with Oblimin rotation. Almost all items loaded on the respective factors of their scales, with minimal cross-loadings of items from the measures of turnover intentions, career commitment, and career advancement motivation (see Supplementary Table E).

Next, a confirmatory factor analysis (CFA) was conducted to obtain a statistical indication of the validity of our measurement model. Again, we tested the model with nine factors, as suggested by the PA. We defined the model such that all items loaded on their respective factors. Because the assumption of multivariate normality was violated, we used Satorra–Bentler test statistics and robust standard errors. The results of the CFA showed that the measurement model did not reach good fit, χ^2^ = 5126.64, *p* < 0.001, *df* = 1238, χ^2^/*df* = 4.14, RMSEA = 0.07, CFI = 0.89, TLI = 0.88. Based on the cross-loadings in the EFA, we deleted two items from the measures, after which our CFA did indicate good fit, χ^2^ = 4490.84, *p* < 0.001, *df* = 1139, χ^2^/*df* = 3.94, RMSEA = 0.07, CFI = 0.90, TLI = 0.90. Accordingly, we used all measures as separate outcome variables. The deleted items were omitted from all analyses.

### Hypothesis Testing

In order to test the first part of our first hypothesis (H1a), we conducted a 2 (deep-level dissimilarity: yes vs. no) × 2 (surface-level dissimilarity: yes vs. no) between-subjects ANOVA, with inclusion as the dependent variable.^4^ The descriptive statistics can be found in Supplementary Table F of the supplement. We obtained a main effect of deep-level dissimilarity, *F*(1, 872) = 46.08, *p* < 0.001, η_p_^2^ = 0.05, which indicated that participants who perceived themselves to be deep-level dissimilar to most others at work scored lower on felt inclusion (*M* = 4.79, *SD* = 1.31) compared to those who perceived themselves to be deep-level similar (*M* = 5.42, *SD* = 0.95). We obtained no main effect of perceived surface-level dissimilarity on inclusion, *F*(1, 872) = 2.99, *p* = 0.084. Furthermore, we obtained no interaction between deep-level dissimilarity and surface-level dissimilarity, *F*(1, 872) = 1.22, *p* = 0.269, suggesting that the influence of perceived deep-level dissimilarity on felt inclusion was not dependent on whether participants perceived themselves to be surface-level dissimilar to most others at work.^5^ These results partially support our hypothesis (H1a), as only deep-level dissimilarity was related to felt inclusion. The analyses of simple effects using Tukey’s HSD procedure indicated that participants who perceived themselves as only deep-level dissimilar scored lower on inclusion (*M* = 4.89, *SD* = 1.05) than those who perceived themselves as similar in both ways (*M* = 5.43, *SD* = 0.95), *t*(872) = 5.52, *p* < 0.001, and also scored lower than those who perceived only surface-level dissimilarity (*M* = 5.37, *SD* = 0.99), *t*(872) = 3.63, *p* = 0.002. Furthermore, participants who perceived themselves as only surface-level dissimilar did not differ in inclusion from those who perceived similarity in both ways, *t*(872) = 0.54, *p* = 0.949. Participants who perceived both deep-level and surface-level dissimilarity scored lower on inclusion (*M* = 4.62, *SD* = 1.74) than those who perceived themselves as similar in both terms, *t*(872) = 5.94, *p* < 0.001, and those who perceived themselves as only surface-level dissimilar, *t*(872) = 4.60, *p* < 0.001. Lastly, there was no difference between participants who perceived themselves as only deep-level dissimilar and those who perceived themselves as both deep-level and surface-level dissimilar, *t*(872) = 1.74, *p* = 0.306.

To test our remaining hypotheses, we initially treated the five dependent variables independently. This means we first tested Hypothesis 1b using a MANOVA. In order to test Hypotheses 2, 3a, and 3b, we conducted mediation, moderation and moderated mediation analyses using PROCESS (Hayes, 2013). The results of these analyses are presented in the Supplementary Materials. For simplicity of presentation, per the suggestion of the editor, here we present results from two structural equation models that capture the five dependent variables in a single latent variable “work-related outcomes.” For these models we used the *lavaan* package in R. To fit parsimonious models, we created item parcels as indicators for all work-related variables except for job satisfaction, because job satisfaction consisted of only three items. Parcels have shown to produce more reliable latent variables than individual items and are particularly useful when the measurement model is not of direct interest (Little et al., 2013), as is the case for us. The models we constructed did not reach good fit, but this is less of a concern for us given that our primary goal was to test our hypotheses using our theoretical structural equation models. Furthermore, as the assumption of multivariate normality was violated, we used robust estimation methods (“MLM” option in *lavaan*) for all analyses.

The first model tested Hypothesis 1b – namely, that dissimilarity would predict work-related outcomes – using a 2 (deep-level dissimilarity: yes vs. no) × 2 (surface-level dissimilarity: yes vs. no) between-subjects ANOVA with the latent variable work-related outcomes as our dependent variable, χ^2^ = 455.23, *p* < 0.001, *df* = 69, χ^2^/*df* = 6.60, RMSEA = 0.09, CFI = 0.90, TLI = 0.88. We obtained a main effect of deep-level dissimilarity, *b* = -0.09, *SE* = 0.04, *p* = 0.017, 95% CI [-0.16; -0.02], which indicated that participants who perceived themselves to be deep-level dissimilar to most others at work scored lower on the work-related outcomes than those who perceived themselves to be deep-level similar. We obtained no main effect of perceived surface-level dissimilarity on work-related outcomes, *b* = 0.03, *SE* = 0.03, *p* = 0.201, 95% CI [-0.02; 0.09].^6^ Furthermore, we obtained no interaction between deep-level dissimilarity and surface-level dissimilarity, *b* = -0.05, *SE* = 0.05, *p* = 0.337, 95% CI [-0.15; 0.05], suggesting that the influence of deep-level dissimilarity on work-related outcomes does not depend on the degree of surface-level dissimilarity. This partially supports our hypothesis (H1b), as only deep-level dissimilarity was related to work-related outcomes.^7^ In order to exploratively assess the simple effects, we used the Bonferroni correction, thus resulting in an adjusted critical value of 0.008. Using this alpha as a criterion, no simple effects reached significance. These analyses can be found in the Supplementary Materials.

**FIGURE 1:**
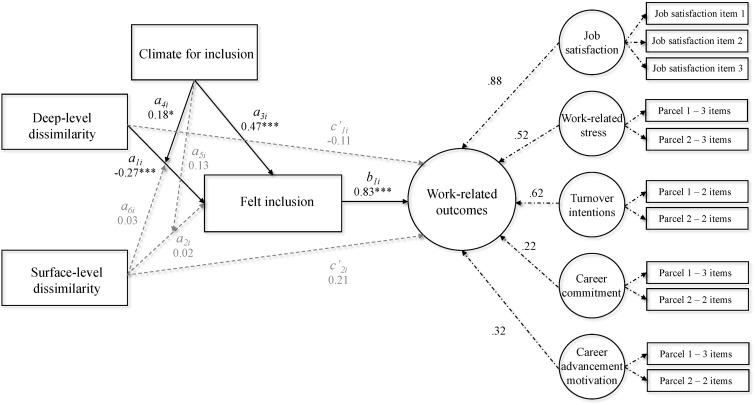
Conceptual overview of the structural equation model with standardized factor loadings and unstandardized parameter estimates. ^∗∗∗^*p* < 0.001, *^∗^p* < 0.05.

The second model tested Hypotheses 2, 3a, and 3b – namely that felt inclusion would mediate the relationship between dissimilarity and work-related outcomes, that a climate for inclusion would moderate the relationship between perceived dissimilarity and felt inclusion and that a climate for inclusion would positively relate to felt inclusion. We used this model with the latent dependent variable “work-related outcomes” (which was indicated by the five dependent variables), one mediator (felt inclusion), one moderator (climate for inclusion), and two independent variables (deep-level and surface-level dissimilarity), χ^2^ = 990.09, *p* < 0.001, *df* = 130, χ^2^/*df* = 7.62, RMSEA = 0.10, CFI = 0.82, TLI = 0.78.^8^ See Figure 1 for a conceptual overview of the current model and Supplementary Tables I, J for the statistics.

Supporting Hypothesis 2, the results indicated that felt inclusion mediated the relationship between perceived deep-level dissimilarity and the work-related outcomes, as shown by the significant indirect relationship, *a*_1_*b*_1_ = -0.22, *p* = 0.001. Perceived surface-level dissimilarity did not have an indirect relationship with work-related outcomes, *a*_2_*b*_1_ = 0.02, *p* = 0.827.

**FIGURE 2:**
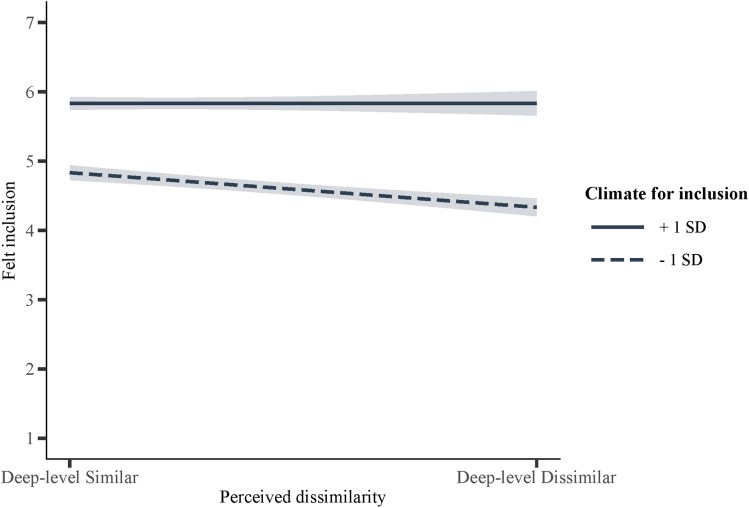
The moderation effect by climate for inclusion on the relationship between deep-level dissimilarity and felt inclusion.

Results furthermore indicated that an inclusive climate can buffer the negative effects of deep-level dissimilarity, *a*_4_ = 0.18, *p* = 0.019, which supports Hypothesis 3a. That is, participants who perceived themselves as deep-level dissimilar to most others at work felt less included compared to those who perceived themselves as deep-level similar when they perceived a negative (-1 *SD*; see Figure 2), *a*_4_ = -0.45, *p* < 0.001, or average (mean), *a*_4_ = -0.27, *p* < 0.001, climate for inclusion. When they perceived a positive climate for inclusion (+1 *SD*), however, participants who perceived themselves as deep-level dissimilar felt equally included as those who perceived themselves as deep-level similar, *a*_4_ = -0.09, *p* = 0.369. In addition, the more positive participants perceived the climate for inclusion to be, the more included they felt. Importantly, while the latter effect was stronger among participants who perceived themselves as deep-level dissimilar, it was also present among participants who perceived themselves as similar to most others at work, reflecting the direct main effect of climate for inclusion on felt inclusion, *a*_3_ = 0.47, *p* < 0.001. Supporting Hypothesis 3b, this suggests that a climate for inclusion is beneficial to all employees. Furthermore, because a positive climate for inclusion (+1 *SD*) buffered the negative relationship between deep-level dissimilarity and felt inclusion, it also neutralized the adverse indirect relationship between perceived deep-level dissimilarity and work-related outcomes, *a*_1_*b*_1_ = -0.08, *p* = 0.375.^9^

## Discussion

Previous research demonstrated a relationship between employee dissimilarity, organizational climate, and inclusion at work. We replicate and extend these findings in two important ways. First, we provide a first examination of the independent and joint effects of surface-level and deep-level dissimilarity on social inclusion, thus extending previous work that has only considered the effect of surface-level dissimilarity (Jansen et al., 2017). We found that perceived deep-level (but not surface-level) dissimilarity is negatively related to felt inclusion. Since no interaction between the two types of dissimilarity was obtained, the relationship between deep-level dissimilarity and felt inclusion does not appear to depend on surface-level dissimilarity. Second, we extend the findings obtained by Jansen et al. (2017) to other work-related outcomes than absenteeism by demonstrating that felt inclusion acts as a mediator between deep-level dissimilarity and participants’ job satisfaction, work-related stress, and turnover intentions. Furthermore, we showed that the negative relationship between perceived deep-level dissimilarity and felt inclusion was buffered by a perceived positive climate for inclusion in a similar way as Jansen et al. (2017) found to be the case for objective gender dissimilarity.

Our finding that only deep-level dissimilarity was related to feelings of inclusion is interesting, considering that most organizational diversity programs (e.g., from 1980–2002 in the United States; Dobbin et al., 2011) tend to focus on surface-level diversity only. Our findings suggest that by also focusing on deep-level dissimilarity in diversity programs, there is a potential for improvement of inclusion in organizations. This finding is also in line with earlier research. For example, Phillips and Loyd (2006) found that people who are only deep-level dissimilar, and not surface-level dissimilar, were less likely to express their deviance because they expected the social disapproval of others over it. The expectation of social disapproval is possibly related to lower feelings of inclusion among those who are deep-level dissimilar.

Furthermore, we found that a positive climate for inclusion is beneficial for the felt inclusion of employees, and consequently for their job satisfaction, work-related stress, turnover intentions, career commitment, and career advancement motivation in the organization. Importantly, a climate for inclusion was found to not only benefit the employees that perceived themselves to be “dissimilar” to most others, but also the ones that perceived themselves to be “similar.” These findings suggest that both minority and majority group members are better off in an organizational climate where people who are dissimilar are being valued and accepted as they are. Majority group members may be positively affected by such a work climate because it affords them the freedom to be different as well. If they wish to deviate from the norm, they would likely still be accepted. Hence, a climate for inclusion enhances feelings of inclusion in the organization – for everyone.

While most of our hypotheses were supported, we also obtained some unexpected results. We expected surface-level dissimilarity to be negatively related to social inclusion, which was indeed reflected in the significant zero-order correlation between surface-level dissimilarity and inclusion (*r* = -0.08, *p* = 0.015). However, this effect disappeared when deep-level dissimilarity was simultaneously taken into account, suggesting that surface-level dissimilarity may only affect inclusion at work to the extent that it is accompanied by a sense of deep-level dissimilarity. Another explanation for the lack of a relationship between surface-level dissimilarity and inclusion is our measurement method, which did not assess the degree of perceived dissimilarity. It is possible that the degree of perceived dissimilarity was lower for those who perceived themselves as surface-level versus deep-level dissimilar. This will be discussed in the limitations section below. A second unexpected finding (reported in our Supplementary Materials) was that surface-level dissimilarity was positively, rather than negatively, related to career commitment and career advancement motivation in the organization. A possible explanation could be that those who perceived themselves to be surface-level dissimilar to others at work are compensating for their dissimilarity through increased motivation and commitment. Indeed, previous research shows that impending discrimination can lead people to distance themselves from stereotypes in order to avoid or overcome the maltreatment (Kaiser and Miller, 2001). If the participants who reported surface-level dissimilarity differed from others on a characteristic that is stereotyped to imply lower career advancement motivation and lower career commitment (e.g., being female; Williams and Dempsey, 2014), then their increased motivation and commitment may have been a form of overcompensation. Another possibility is that these participants are not more motivated or committed in order to compensate for a stereotyped group image, but in order to level the playing field because being equally motivated and committed as majority employees would not help them get ahead.

### Practical Implications

In this research we observed that feelings of inclusion are an important factor in the negative relationship between deep-level dissimilarity and work outcomes. This suggests that in order to limit or buffer the negative effects of dissimilarity, organizations might focus on improving employees’ sense of inclusion. Doing so would likely benefit both individual outcomes (e.g., the well-being of employees) as organizational outcomes (e.g., lower turnover intentions and higher commitment of their employees). This study can potentially inspire organizations to develop and implement more effective diversity policies by focusing on the inclusion of all employees – including those who are not visibly different from others. Notwithstanding these conclusions, it is important to note that the effect sizes in our study are relatively small. While perceived dissimilarity and felt inclusion seem to be important factors in the workplace, the modest effect sizes show that a stronger sense of inclusion is not a miracle cure for work-related issues. Nonetheless, according to our results, a climate for inclusion is something worth striving toward if one wants to improve the well-being and performance of employees.

A first step in improving the organizational climate for inclusion entails a shift from a one-sided focus on surface-level differences between employees to also integrating deep-level differences in their diversity management strategies. For example, in addition to implementing policies that focus on those who are surface-level dissimilar to the majority of employees, such as special programs for women or ethnic minorities, organizations could also consider ways to make those who are deep-level dissimilar (those with different personalities, preferences, or perspectives) feel included. For instance, organizations could benefit from actively inviting minority perspectives, communicating the worth of all employees, or establishing employee networks for groups that may be less visibly different from the norm (e.g., for LGBT + employees).

Specifically, in prior work three dimensions have been outlined that need to be considered by organizations striving toward a climate for inclusion (Nishii and Rich, 2013). The first dimension, which lays the groundwork for the two other dimensions, focuses on establishing a “level playing field.” Making practices to combat unfair and biased actions visible to all employees will send a signal about intolerance of discrimination in the organization. Second, organizations should have an integration strategy that facilitates inclusion of all individuals in the workplace. As evident from our results, dissimilarity is negatively related to inclusion. An integration strategy is necessary in order to ensure that employees do not feel pressured to assimilate into the dominant culture, as there are many indications that being one’s authentic self fosters one’s well-being and performance (Thomaes et al., 2017; Schmader and Sedikides, 2018), while hiding or constraining one’s identity undermines these outcomes (Hewlin, 2003; Ellemers and Barreto, 2006). Third, decision-making should be inclusive. This ensures that perspectives from employees who have not traditionally been involved in the decision-making are also heard and incorporated in the process. Sharing and integrating knowledge of everyone not only gives a voice to all employees, but also results in more creativity (Men et al., 2017).

### Limitations and Future Research

There are several potential limitations of this study that could be resolved in future research. A first issue regards our assessment of perceived dissimilarity. We utilized a top-down method of defining surface-level and deep-level dissimilarity by asking participants whether they felt visibly or invisibly dissimilar, while providing some examples of the two dimensions. This has the limitation that we cannot be sure that participants agreed with our typology (e.g., that gender and ethnicity could be considered surface-level characteristics), and which specific characteristic participants had in mind when they indicated feeling dissimilar. For example, we do not know whether participants felt different from others in terms of their personality traits, their values, or their sexual orientation.

Furthermore, we chose to use a single dichotomous item for each type of dissimilarity because we wanted to clearly distinguish between employees who perceive themselves as dissimilar and employees who perceive themselves as similar to most others at work. This way, there would be no doubt that the participants intended to categorize themselves as dissimilar or similar. The disadvantage of using dichotomous items, however, is that we do not know what the degree of perceived dissimilarity is. This information could be important, as it may be that inclusion might be affected only by a certain degree of dissimilarity.

The disadvantage of using single items is that single-item measures have lower reliability and validity compared to multi-item scales (Diamantopoulos et al., 2012). Another disadvantage of using single items is that we only have an indication of dissimilarity in a general sense, namely dissimilarity compared to most others at work. However, this doesn’t allow us to differentiate the extent to which they feel dissimilar in subcontexts, such as relative to one’s team members, supervisors or support staff. It is possible that the strength of the relationship between dissimilarity and inclusion differs per context. For instance, it may be possible that this relationship is stronger within one’s team than in the office in general, as interdependence may be stronger in the former than the latter context.

Future studies addressing perceived dissimilarity at work could use multi-item and continuous measures of dissimilarity in order to understand the influence of the degree of dissimilarity and the significance of dissimilarity in different contexts. For the purposes of the current study, knowing whether participants perceived themselves as surface-level and/or deep-level dissimilar from others was the most important. We also note that using single items, as we have done, is not necessarily worse than using multi-item scales (Gardner et al., 1998).

Future research could, furthermore, use a bottom-up method of defining dissimilarity in order to examine more in-depth exactly what it is that makes employees feel dissimilar. Participants could indicate in what exact ways they feel dissimilar and whether they categorize these under surface- or deep-level dissimilarity. This would allow a more fine-grained analysis as to how dissimilarity on the basis of specific characteristics affects social inclusion and what patterns can be discerned. For instance, it would be interesting to investigate whether dissimilarity in characteristics indicating a stigmatized status (e.g., skin color, gender, or wearing the hijab) would be as negatively related to felt inclusion as dissimilarity in characteristics indicating non-stigmatized status. This is an interesting issue to explore in future research. Furthermore, there is some indication that gender and ethnicity might differentially affect the two subdimensions of social inclusion, authenticity, and belonging. Namely, women in engineering experience pressure to play down their female identity (Faulkner, 2011), whereas African American students experience social exclusion (Strayhorn, 2008). Hence, the first may experience a lowered sense of inclusion through lowered authenticity and the latter through lowered belonging. It is also important to keep in mind that people may feel dissimilar in multiple ways at the same time (e.g., as a Black woman in a workplace in which White men are the majority), which might open ways to multiple disadvantages for one person. More research is needed to understand how dissimilarity in intersectional terms affects people, as it is not only theoretically relevant, but also reflects the reality in which people belong to multiple categories at the same time (Cole, 2009).

Although our CFA indicated good fit of the measurement model, our SEM models did not reach good fit. This means that we did not specify all the important relationships that the data suggests. We decided not to increase model fit by adding residual correlations or covariances between our latent variables based on the modification indices, since doing so does not add anything to the theoretical model that we wanted to test. However, it does mean that we do not yet fully understand the relationships between job satisfaction, work-related stress, turnover intentions, career commitment, and career advancement motivation within the organization. As this was not the scope of the current paper, we did not investigate this, but it is important to do so more systematically in future research.

Furthermore, as is the convention in organizational surveys, participants received the demographic questions first, including whether they perceived themselves as dissimilar to their colleagues. This could have made their dissimilarity salient and may have influenced their answers to the questions that followed. However, one could argue that this reflects the reality of situations in which people are addressed in terms of their demographic characteristics, and tend to be chronically aware of their minority status (Kim-Ju and Liem, 2003).

Lastly, research is needed to uncover what organizations can do to create and maintain a climate for inclusion at work. Even though previous research has described the characteristics of a climate for inclusion (e.g., Nishii and Rich, 2013), which policies organizations can implement to develop such a climate, or to prevent it from deteriorating over time, has not yet been examined. As the current study highlights the importance of a climate for inclusion for people who perceive themselves as deep-level dissimilar, longitudinal studies that focus on conditions that foster the development of such a climate can offer an important next step toward creating more inclusive workplaces.

## Conclusion

In summary, the research reported in this contribution demonstrates that subjective perceptions of dissimilarity and the extant climate for inclusion relate to employees’ feelings of inclusion in important ways. Our results, furthermore, suggest that deep-level dissimilarity is an important factor in the processes that are at work in diverse groups, even more so than surface-level dissimilarity. More research is needed to pinpoint which surface-level or deep-level characteristics in particular are at play in this process and to understand how a climate for inclusion can be realized in order to create and maintain inclusive workplaces.

## Data Availability

The informed consent stated that the data of participants would not be shared with third parties. Therefore, our data cannot be made publicly available. Requests for access to the dataset should be directed to NE, n.ellemers@uu.nl.

## Author Contributions

OŞ conducted the analyses. OŞ, JvdT, WJ, and NE wrote the drafts of the manuscript. EB read the drafts of the manuscript and provided feedback. JvdT, WJ, EB, and NE developed the study. NE secured access to the organization and obtained permission to conduct this study. EB obtained approval from the ethical committee and collected the data.

## Conflict of Interest Statement

The authors declare that the research was conducted in the absence of any commercial or financial relationships that could be construed as a potential conflict of interest.

## Footnotes

1 One of the objectives of this study was to validate our measure of the perceived climate for inclusion. Our survey thereto included additional measures that assessed the perceived diversity climate (Hobman et al., 2004), the perceived inclusivity of the organizational culture (Ashikali and Groeneveld, 2015), interpersonal justice (Colquitt, 2001), and social desirability (Rudmin, 1999).

2 Contrary to Hobman’s approach (2004), we chose to use a single dichotomous item for each type because we wanted to clearly distinguish between employees who perceive themselves as dissimilar and employees who perceive themselves as similar to most others at work. This way, there would be no doubt that the participants intended to categorize themselves as dissimilar or similar. The implications of this choice are further discussed in the Section “Limitations and Future Research.”

3 For employees who perceive themselves as dissimilar, both the measure of climate for inclusion and the measure of felt inclusion tap into how employees who are dissimilar are treated at work. In contrast, for employees who perceive themselves as similar, there is a difference between the two measures, as felt inclusion does not tap into the treatment of someone who is dissimilar. This might raise the question whether climate for inclusion and felt inclusion are different constructs for those who perceive themselves as dissimilar. To answer this question, we tested whether there was a distinction between perceived climate for inclusion and felt inclusion for both employees who perceived themselves as similar or dissimilar (surface-level and/or deep-level). We first performed a PA to determine the number of significant factors, which resulted in four factors. Afterward, we conducted two EFAs, using principal axis factoring with Oblimin rotation and only retained factor loadings that exceeded 0.30. The results were similar for participants who perceived themselves as similar and dissimilar, in that all items of perceived climate for inclusion loaded on a single factor and the items of felt inclusion loaded on the three remaining factors (see Supplementary Tables C, D). This is in line with Jansen et al. (2014) who found that items for the subdimensions authenticity and belonging loaded on separate factors.

4 This analysis was repeated after removal of outliers (+3 SD), yielding similar results. Furthermore, in the Supplementary Materials, we report an ANCOVA, which we conducted to test the main and interactive effects of deep-level and surface-level dissimilarity on inclusion, while controlling for sex, age, education level, tenure, senior position and junior position, yielding similar results.

5 We also examined whether perceived (in)visible dissimilarity differentially influenced felt belonging and felt authenticity, the two subdimensions of inclusion. These analyses can be found in the Supplementary Materials.

6 The regular MANOVA presented in the Supplementary Materials, with job satisfaction, work-related stress, turnover intentions, career commitment, and career advancement motivation within the organization as separate dependent variables showed that deep-level dissimilarity predicted the first three work-related outcomes, but not career commitment and career advancement motivation. In contrast, surface-level dissimilarity only predicted career commitment and career advancement motivation. Interestingly, participants who perceived surface-level dissimilarity (vs. similarity) scored higher on these outcomes than.

7 This analysis was repeated after removal of outliers (+3 SD), yielding similar results.

8 The moderated mediation analyses using PROCESS, where a separate moderated mediation was tested for each of the five dependent variables, are described in the Supplementary Materials.

9 This analysis was repeated after removal of outliers (+3 SD), yielding similar results.
