# Supplementary material for: Looking Beyond Our Similarities: How Perceived (In)Visible Dissimilarity Relates to Feelings of Inclusion at Work
Source: Front Psychol. 2019 Mar 27;10:575. doi: 10.3389/fpsyg.2019.00575 (PMC6445863; doi:10.3389/fpsyg.2019.00575)
Supplement: Supplementary file 1 [file Table_1.docx]

Supplementary Material

Looking Beyond Our Similarities: How Perceived (In)Visible Dissimilarity Relates to Feelings of Inclusion at Work

Onur Şahin*, Jojanneke van der Toorn, Wiebren S. Jansen, Edwin J. Boezeman, Naomi Ellemers

*** Correspondence:** Corresponding Author: o.sahin@uu.nl

# Additional Analyses

## Authenticity vs. belonging

Since we can distinguish between feelings of authenticity and feelings of belonging as dimensions of felt inclusion (see Jansen, Otten, van der Zee, & Jans, 2014), we explored whether the employees who felt deep-level or surface-level dissimilar differentially experienced authenticity and belonging. Furthermore, we explored whether there were differences between authenticity and belonging within participants who perceived themselves as similar or dissimilar. To this end, we conducted a mixed design ANOVA, in which deep-level and surface-level dissimilarity were the between-subjects factors and the inclusion dimension (authenticity vs. belonging) was the within-subjects factor, resulting in a 2 (deep-level dissimilarity: yes vs. no) x 2 (surface-level dissimilarity: yes vs. no) x 2 (inclusion-facet: authenticity vs. belonging) design, with authenticity and belonging as the outcome variables and deep-level and surface-level dissimilarity as predictors. We obtained a significant main effect for inclusion, *F*(1, 872) = 135.84, *p* < .001, *η_p_²*  = .135, indicating that participants on average scored lower on authenticity than on belonging (*M* = 5.07, *SD* = 1.19 vs. *M* = 5.46, *SD* = 1.11). Furthermore, we obtained a significant main effect for deep-level dissimilarity, *F*(1, 872) = 46.07, *p* < .001, *η_p_²*  = .050, but not for surface-level dissimilarity, *F*(1, 872) = 2.99, *p* = .084, *η_p_²*  = .003, indicating that participants who perceived deep-level dissimilarity scored lower on both dimensions of inclusion than those who perceived deep-level similarity, (*M* = 4.79, *SD* = 1.31 vs. *M* = 5.42, *SD* = 0.95).

We obtained no significant interaction between deep-level and surface-level dissimilarity, *F*(1, 872) = 1.23, *p* = .269, *η_p_²*  = .001, and no significant interaction between surface-level dissimilarity and inclusion, *F*(1, 872) = 2.31, *p* = .129, *η_p_²*  = .003, but we did obtain a significant interaction between deep-level dissimilarity and inclusion, *F*(1, 872) = 8.44, *p* = .004, *η_p_²* = .010. This interaction indicates that the difference between authenticity and belonging was bigger for participants who perceived deep-level dissimilarity (*M_authenticity_* = 4.55, *SD_authenticity_* = 1.42, *M_belonging_* = 5.03, *SD_belonging_* = 1.36) compared to participants who perceived deep-level similarity (*M_authenticity_* = 5.24, *SD_authenticity_* = 1.05, *M_belonging_* = 5.60, *SD_belonging_* = 0.99). These findings suggest that while all employees experienced less authenticity than belonging, those who perceived themselves as deep-level dissimilar experienced particularly low authenticity.

Even though there was no three-way interaction, we exploratively examined its simple effects using Tukey’s HSD procedure. The results indicated that the deep-level x surface-level dissimilarity interaction effects were mostly similar for authenticity and belonging (see Table K for the group means). Participants who perceived both deep-level and surface-level dissimilarity did not differ from those who perceived only deep-level dissimilarity on both authenticity, *t*(872) = 1.50, *p* = .437, and belonging, *t*(872) = 1.78, *p* = .284. However, participants who perceived both types of dissimilarity did score lower than those who perceived similarity in both terms on both authenticity, *t*(872) = 5.74, *p* < .001, and belonging, *t*(872) = 5.45, *p* < .001, and also scored lower than those who perceived only surface-level dissimilarity on authenticity, *t*(872) = 4.97, *p* < .001, and belonging, *t*(872) = 3.67, *p* = .002. Lastly, although participants who perceived only deep-level dissimilarity scored lower on authenticity than those who perceived only surface-level dissimilarity, *t*(872) = 4.35, *p* < .001, they did not score lower on belonging, *t*(872) = 2.43, *p* = .072. This pattern of findings suggests that deep-level dissimilarity and surface-level differ mostly in terms of authenticity, as they did not differ in terms of belonging.

## Do covariates affect the results of the main analyses?

In order to control for the possible influence of covariates on the relationship between deep-level and surface-level dissimilarity, and inclusion, we conducted a 2 (deep-level dissimilarity: yes vs. no) x 2 (surface-level dissimilarity: yes vs. no) ANCOVA with inclusion as the dependent variable, including participant sex, (male =1 vs. female = 0), age (centered), education (centered, elementary = 1, secondary = 2, lower vocational = 3, middle vocational = 4, higher professional = 5 and university = 6), tenure (centered, in years) and position (senior = 1 vs. not senior = 0; junior = 1 vs. not junior = 0) as covariates. After controlling for the covariates, we obtained a significant main effect of deep-level dissimilarity, *F*(1, 716) = 34.28, *p* < .001, *η_p_*^2^ = .05, but no effect of surface-level dissimilarity on inclusion, *F*(1, 716) = 1.58, *p* = .209, *η_p_*^2^ < .01, and no interaction between deep-level dissimilarity and surface-level dissimilarity, *F*(1, 716) = 0.76, *p =* .383, *η_p_*^2^ < .01 These results are similar to the main analyses reported in the manuscript.

The covariates age, *F*(1, 716) = 5.70, *p =* .017, *η_p_*^2^ = .01, and education *F*(1, 716) = 8.66, *p =* .003, *η_p_*^2^ = .01 also significantly predicted inclusion, indicating that as age increased, people felt less included, *b* = -0.11. In contrast, as education increased, people felt more included, *b* = 0.13. No significant effects were obtained for participant sex, *F*(1, 716) = 2.65, *p =* .104, *η_p_*^2^ < .01, tenure, *F*(1, 716) = 3.54, *p =* .060, *η_p_*^2^ < .01, whether they were a senior, *F*(1, 716) = 0.73, *p =* .392, *η_p_*^2^ < .01, or junior, *F*(1, 716) = 0.11, *p =* .732, *η_p_*^2^ < .01.

## MANOVA on all observed dependent variables

In addition to the analyses reported in the main text, we also conducted more fine-grained analyses to test Hypothesis 1b, namely whether dissimilarity relates negatively to the work-related outcomes. We conducted a 2 (deep-level dissimilarity: yes vs. no) x 2 (surface-level dissimilarity: yes vs. no) MANOVA with job satisfaction, work-related stress, turnover intentions, career commitment and career advancement motivation within the organization as dependent variables.^[[1]](#footnote-1)^ A summary of the statistics of these analyses can be found in Table F. We obtained a significant multivariate effect for both perceived deep-level, *F*(5, 866) = 6.30, *p* < .001, *η_p_^2^* = .04, and perceived surface-level dissimilarity on the dependent variables, *F*(5, 866) = 2.82, *p* = .015, *η2* = .02; however, no significant interaction was obtained, *F*(5, 866) = 1.14, *p* = .336. The univariate tests showed that perceived deep-level dissimilarity predicted job satisfaction, *F*(1, 871) = 21.58, *p* < .001, *η_p_^2^* = .02, work-related stress, *F*(1, 871) = 17.44, *p* < .001, *η_p_^2^* = .02, and turnover intentions, *F*(1, 872) = 7.89, *p* = .005, *η_p_^2^* = .01. Consistent with our hypotheses, those who perceived themselves as deep-level dissimilar scored lower than those who perceived themselves as deep-level similar on job satisfaction (*M* = 5.39, *SD* = 1.19 vs. *M* = 5.77, *SD* = 0.98), lower on work-related stress (indicating more stress; *M* = 3.80, *SD* = 1.02 vs. *M* = 4.08, *SD* = 0.90) and lower on turnover intentions (indicating more turnover intentions; *M* = 0.58, *SD* = 0.36 vs. *M* = 0.66, *SD* = 0.33). Perceived deep-level dissimilarity did not significantly predict career commitment and career advancement motivation. The univariate tests, furthermore, showed that perceived surface-level dissimilarity did predict career commitment, *F*(1, 872) = 11.54, *p* = .001, *η_p_^2^* = .01, and career advancement motivation, *F*(1, 872) = 5.64, *p* = .018, *η_p_^2^* = .01, but not in the hypothesized direction. Contrary to our hypotheses, participants who perceived surface-level dissimilarity scored higher than those who perceived surface-level similarity on career commitment (*M* = 5.06, *SD* = 1.17 vs. *M* = 4.78, *SD* = 1.04) and higher on career advancement motivation (*M* = 4.59, *SD* = 0.93 vs. *M* = 4.42, *SD* = 0.85) .

Even though there was no interaction, we exploratively examined^[[2]](#footnote-2)^ the simple effects using Tukey’s HSD procedure. Results indicated that while participants who perceived both deep-level and surface-level dissimilarity did not differ from those who perceived only deep-level dissimilarity on job satisfaction, *t*(871) = 0.49, *p* = .962, they did score lower on job satisfaction than those who perceived similarity in both terms, *t*(871) = 3.03, *p* = .013, and also scored lower than those who perceived only surface-level dissimilarity, *t*(871) = 3.35, *p* = .005 (See Table F for the means). Furthermore, participants who perceived only deep-level dissimilarity scored lower on job satisfaction than those who perceived only surface-level dissimilarity, *t*(871) = 3.55, *p* = .002. For work-related stress, participants who perceived only deep-level dissimilarity did not differ from those who perceived only surface-level dissimilarity, *t*(871) = 2.07, *p* = 0.163, and those who perceived both types of dissimilarity, *t*(871) = 2.09, *p* = .156. In contrast, participants who perceived both types of dissimilarity had a lower score on stress (indicating more stress) than those who perceived only surface-level dissimilarity, *t*(871) = 3.68, *p* = .001, and those who perceived similarity in both terms, *t*(871) = 3.81, *p* < .001. Interestingly, the only significant main effect for turnover intentions was a lower score (indicating more turnover intentions) among participants who perceived only deep-level dissimilarity than among participants who perceived both types of similarity, *t*(872) = 2.79, *p* = .028. For career commitment, the only significant effect was a higher score among participants who perceived only surface-level dissimilarity than participants who perceived only deep-level dissimilarity, *t*(872) = 3.46, *p* = .003. Lastly, no differences between groups were found for career advancement motivation.

## Simple effects of the interaction between deep-level and surface-level dissimilarity on the latent variable work-related outcomes

In order to exploratively probe the interaction we tested between deep-level and surface-level dissimilarity on the latent variable ‘work-related outcomes’ in the main text, we conducted Bonferroni corrected tests, setting our alpha at .008 to account for the six comparisons. The analyses indicated that participants who perceived themselves as only deep-level dissimilar did not differ on inclusion from those who perceived themselves as similar in both ways, *p* = .020, or from those who perceived only surface-level dissimilarity, *p* = .053, or from those who perceived dissimilarity in both ways, *p* = .591. Furthermore, participants who perceived themselves as only surface-level dissimilar did not differ in inclusion from those who perceived similarity in both ways, *p* = .202, or from those who perceived both types of dissimilarity, *p* = .591. Lastly, participants who perceived dissimilarity in both ways did not differ from those who perceived similarity in both ways, *p* = .040.

## Moderated mediation analyses with the five work-related outcomes as separate variables

In the main text we reported a test of Hypothesis 2, 3a and 3b using a model with the dependent latent variable ‘work-related outcomes’, which is indicated by job satisfaction, work-related stress, turnover intentions, career commitment and career advancement motivation. Below we report the analyses with the five work-related outcomes as separate dependent variables, using the PROCESS macro for SPSS (Hayes, 2013). We conducted the analyses only with deep-level dissimilarity as the independent variable, because our results in the main text showed that only deep-level dissimilarity predicted feelings of inclusion.^[[3]](#footnote-3)^ We included feelings of inclusion as the mediator and climate for inclusion as the moderator in all analyses. All analyses were conducted using 5000 bootstrap samples.

In order to test Hypothesis 2, namely that felt inclusion mediates the relationships between perceived dissimilarity and work-related outcomes, we conducted five mediation analyses (PROCESS model 4), each with a different dependent variable. Supporting our hypothesis, each analysis indicated that there was a negative relationship between deep-level dissimilarity and felt inclusion, *b* = -0.59, *p* < .001. When controlling for felt inclusion, there were no direct relationships between deep-level dissimilarity and job satisfaction, *b* = -0.07, *p* = .318, work-related stress, *b* = -0.08, *p* = .259, turnover intentions, *b* = -0.05, *p* = .082, career commitment, *b* = -0.14, *p* = .131, and career advancement motivation, *b* = -0.08, *p* = .283. However, there were indirect effects, mediated through felt inclusion, between perceived deep-level dissimilarity and job satisfaction, *b* = -0.32, 95% CI [-0.44; -0.21], work-related stress, *b* = -0.19, 95% CI [-0.26; -0.12], turnover intentions, *b* = -0.04, 95% CI [-0.06; -0.02], career commitment, *b* = -0.06, 95% CI [-0.13; -0.01], and career advancement motivation, *b* = -0.14, 95% CI [-0.21; -0.09].

In order to test Hypotheses 3a and 3b, namely whether climate for inclusion moderated the relationship between deep-level dissimilarity and felt inclusion (H3a) and whether climate for inclusion positively relates to felt inclusion (H3b), we conducted a moderation analysis (PROCESS model 1). Supporting Hypothesis 3a, the negative relationship between deep-level dissimilarity and felt inclusion was moderated by climate for inclusion, as shown by the interaction between deep­-level dissimilarity and climate for inclusion, *b* = 0.27, *p* < .001. That is, participants who perceived themselves as deep-level dissimilar to most others at work felt less included compared to those who perceived themselves as deep-level similar when they perceived a negative (-1 SD; *b* = -0.53, *p* < .001) or average (mean; *b* = -0.26, *p* < .001) climate for inclusion. In a positive climate for inclusion (+1 SD), participants who perceived themselves as dissimilar to most others at work felt as included as those who perceived themselves as similar*,* *b* < 0.01, *p* = .966 (see Figure 2 in the main text). Supporting Hypothesis 3b, the analysis also showed that the more positive participants perceived the climate for inclusion to be, the more included they felt. Importantly, while the latter effect was stronger among participants who perceived themselves as deep-level dissimilar (as indicated by the interaction effect), it was also present among participants who perceived themselves as similar to most others at work, reflecting the direct main effect of climate for inclusion on felt inclusion*,* *b* = .55, *p* < .001. This suggests that a climate for inclusion is beneficial to all employees.

In addition, we tested the conditional indirect effects by conducting moderated mediation analyses (see Tables G and H for summaries of the statistics). We conducted five separate analyses, each with a different dependent variable (PROCESS model 7). The results indicated that there were only indirect effects, through felt inclusion, of deep-level dissimilarity on job satisfaction, *b* = -0.13, 95% CI [-0.22; -0.06], work-related stress, *b* = -0.08, 95% CI [-0.13; -0.04], turnover intentions, *b* = -0.02, 95% CI [-0.03; -0.01], career commitment, *b* = -0.03, 95% CI [-0.06; -0.01], and career advancement motivation, *b* = -0.06, 95% CI [-0.10; -0.03]. There were no indirect effects of surface-level dissimilarity on any of the work-related outcomes.

Furthermore, because a positive climate for inclusion (+1 SD) buffered the negative relationship between deep-level dissimilarity and feelings of inclusion, it also neutralized the negative indirect relationship between perceived deep-level dissimilarity and job satisfaction, *b* < 0.01, 95% CI [-0.11; 0.11], work-related stress, *b* < 0.01, 95% CI [-0.13; 0.04], turnover intentions, *b* < 0.01, 95% CI [-0.01; 0.01], career commitment, *b* < 0.01, 95% CI [-0.02; 0.02], and career advancement motivation, *b* < 0.01, 95% CI [-0.05;0.05].

Supplementary Figures and Tables

*Table A.* Factor Loadings of the Exploratory Factor Analysis on the Perceived Group Inclusion Scale Using a One-Factor Solution (Principal Axis Factoring, Direct Oblimin Rotation, Factor Loadings > .30)

| Item: “People at work… | Factor 1 |
| --- | --- |
| …give me the feeling that I belong.” | .85 |
| …give me the feeling that I am part of this group.” | .86 |
| …give me the feeling that I fit in.” | .87 |
| …treat me as an insider.” | .81 |
| …like me.” | .85 |
| …appreciate me.” | .80 |
| …are pleased with me.” | .84 |
| …care about me.” | .79 |
| …allow me to be authentic.” | .88 |
| …allow me to be who I am.” | .88 |
| …allow me to express my authentic self.” | .85 |
| …allow me to present myself the way I am.” | .88 |
| …encourage me to be authentic.” | .80 |
| …encourage me to be who I am.” | .81 |
| …encourage me to express my authentic self.” | .78 |
| …encourage me to present myself the way I am.” | .81 |
| Eigenvalue | 11.22 |

*Table B.* Factor Loadings of the Exploratory Factor Analysis on the Perceived Group Inclusion Scale Using a Four-Factor Solution (Principal Axis Factoring, Direct Oblimin Rotation, Factor Loadings > .30)

| Item: | Factor 1 | Factor 2 | Factor 3 | Factor 4 |
| --- | --- | --- | --- | --- |
| “People at work…give me the feeling that I belong.” | .82 |  |  |  |
| …give me the feeling that I am part of this group.” | 1.02 |  |  |  |
| …give me the feeling that I fit in.” | .92 |  |  |  |
| …treat me as an insider.” | .75 |  |  |  |
| …like me.” |  | .96 |  |  |
| …appreciate me.” |  | .81 |  |  |
| …are pleased with me.” |  | .91 |  |  |
| …care about me.” |  | .63 |  |  |
| …allow me to be authentic.” |  |  | .95 |  |
| …allow me to be who I am.” |  |  | .94 |  |
| …allow me to express my authentic self.” |  |  | .84 |  |
| …allow me to present myself the way I am.” |  |  | .99 |  |
| …encourage me to be authentic.” |  |  |  | .94 |
| …encourage me to be who I am.” |  |  |  | .96 |
| …encourage me to express my authentic self.” |  |  |  | .96 |
| …encourage me to present myself the way I am.” |  |  |  | .95 |
| Eigenvalue | 3.76 | 3.69 | 3.48 | 3.14 |

*Table C.* Factor Loadings of the Exploratory Factor Analysis on the Perceived Group Inclusion Scale and Perceived Climate for Inclusion Scale Using a Four-Factor Solution for Participants who Perceive Themselves as Similar to Others (Principal Axis Factoring, Oblimin Rotation, Factor Loadings > .30)

| Item: | Factor 1 | Factor 2 | Factor 3 | Factor 4 |
| --- | --- | --- | --- | --- |
| “People at work…give me the feeling that I belong.” |  | .80 |  |  |
| …give me the feeling that I am part of this group.” |  | .80 |  |  |
| …give me the feeling that I fit in.” |  | .83 |  |  |
| …treat me as an insider.” |  | .79 |  |  |
| …like me.” |  | .85 |  |  |
| …appreciate me.” |  | .84 |  |  |
| …are pleased with me.” |  | .88 |  |  |
| …care about me.” |  | .82 |  |  |
| …allow me to be authentic.” |  |  |  | .91 |
| …allow me to be who I am.” |  |  |  | .92 |
| …allow me to express my authentic self.” |  |  |  | .82 |
| …allow me to present myself the way I am.” |  |  |  | .92 |
| …encourage me to be authentic.” |  |  | .93 |  |
| …encourage me to be who I am.” |  |  | .97 |  |
| …encourage me to express my authentic self.” |  |  | .92 |  |
| …encourage me to present myself the way I am.” |  |  | .95 |  |
| Climate for inclusion 1 | .75 |  |  |  |
| Climate for inclusion 2 | .85 |  |  |  |
| Climate for inclusion 3 | .85 |  |  |  |
| Climate for inclusion 4 | .87 |  |  |  |
| Climate for inclusion 5 | .78 |  |  |  |
| Climate for inclusion 6 | .84 |  |  |  |
| Climate for inclusion 7 | .74 |  |  |  |
| Climate for inclusion 8 | .79 |  |  |  |
| Climate for inclusion 9 | .79 |  |  |  |
| Climate for inclusion 10 | .73 |  |  |  |
| Climate for inclusion 11 | .71 |  |  |  |
| Climate for inclusion 12 | .63 |  |  |  |
| Eigenvalue | 7.43 | 5.83 | 3.82 | 3.69 |

*Table D.* Factor Loadings of the Exploratory Factor Analysis on the Perceived Group Inclusion Scale and Perceived Climate for Inclusion Scale Using a Four-Factor Solution for Participants who Perceive Themselves as Dissimilar to Others (Principal Axis Factoring, Oblimin Rotation, Factor Loadings > .30)

| Item: | Factor 1 | Factor 2 | Factor 3 | Factor 4 |
| --- | --- | --- | --- | --- |
| “People at work…give me the feeling that I belong.” |  | .84 |  |  |
| …give me the feeling that I am part of this group.” |  | .84 |  |  |
| …give me the feeling that I fit in.” |  | .84 |  |  |
| …treat me as an insider.” |  | .81 |  |  |
| …like me.” |  | .92 |  |  |
| …appreciate me.” |  | .91 |  |  |
| …are pleased with me.” |  | .89 |  |  |
| …care about me.” |  | .84 |  |  |
| …allow me to be authentic.” |  |  | .93 |  |
| …allow me to be who I am.” |  |  | .85 |  |
| …allow me to express my authentic self.” |  |  | .94 |  |
| …allow me to present myself the way I am.” |  |  | .90 |  |
| …encourage me to be authentic.” |  |  |  | .84 |
| …encourage me to be who I am.” |  |  |  | .98 |
| …encourage me to express my authentic self.” |  |  |  | .89 |
| …encourage me to present myself the way I am.” |  |  |  | .89 |
| Climate for inclusion 1 | .79 |  |  |  |
| Climate for inclusion 2 | .82 |  |  |  |
| Climate for inclusion 3 | .86 |  |  |  |
| Climate for inclusion 4 | .91 |  |  |  |
| Climate for inclusion 5 | .86 |  |  |  |
| Climate for inclusion 6 | .88 |  |  |  |
| Climate for inclusion 7 | .82 |  |  |  |
| Climate for inclusion 8 | .83 |  |  |  |
| Climate for inclusion 9 | .82 |  |  |  |
| Climate for inclusion 10 | .78 |  |  |  |
| Climate for inclusion 11 | .76 |  |  |  |
| Climate for inclusion 12 | .67 |  |  |  |
| Eigenvalue | 8.38 | 6.41 | 3.76 | 3.71 |

*Table E.* Factor Loadings of the Exploratory Factor Analysis on all Variables Using a Nine-Factor Solution (Principal Axis Factoring, Oblimin Rotation, Factor Loadings > .30)

| Item: | 1 | 2 | 3 | 4 | 5 | 6 | 7 | 8 | 9 |
| --- | --- | --- | --- | --- | --- | --- | --- | --- | --- |
| “People at work…give me the feeling that I belong.” |  | .82 |  |  |  |  |  |  |  |
| …give me the feeling that I am part of this group.” |  | .84 |  |  |  |  |  |  |  |
| …give me the feeling that I fit in.” |  | .85 |  |  |  |  |  |  |  |
| …treat me as an insider.” |  | .82 |  |  |  |  |  |  |  |
| …like me.” |  | .88 |  |  |  |  |  |  |  |
| …appreciate me.” |  | .85 |  |  |  |  |  |  |  |
| …are pleased with me.” |  | .89 |  |  |  |  |  |  |  |
| …care about me.” |  | .81 |  |  |  |  |  |  |  |
| …allow me to be authentic.” |  |  |  |  |  | .81 |  |  |  |
| …allow me to be who I am.” |  |  |  |  |  | .81 |  |  |  |
| …allow me to express my authentic self.” |  |  |  |  |  | .71 |  |  |  |
| …allow me to present myself the way I am.” |  |  |  |  |  | .85 |  |  |  |
| …encourage me to be authentic.” |  |  |  |  | .89 |  |  |  |  |
| …encourage me to be who I am.” |  |  |  |  | .90 |  |  |  |  |
| …encourage me to express my authentic self.” |  |  |  |  | .89 |  |  |  |  |
| …encourage me to present myself the way I am.” |  |  |  |  | .88 |  |  |  |  |
| Climate for inclusion 1 | .77 |  |  |  |  |  |  |  |  |
| Climate for inclusion 2 | .85 |  |  |  |  |  |  |  |  |
| Climate for inclusion 3 | .86 |  |  |  |  |  |  |  |  |
| Climate for inclusion 4 | .90 |  |  |  |  |  |  |  |  |
| Climate for inclusion 5 | .82 |  |  |  |  |  |  |  |  |
| Climate for inclusion 6 | .87 |  |  |  |  |  |  |  |  |
| Climate for inclusion 7 | .78 |  |  |  |  |  |  |  |  |
| Climate for inclusion 8 | .82 |  |  |  |  |  |  |  |  |
| Climate for inclusion 9 | .82 |  |  |  |  |  |  |  |  |
| Climate for inclusion 10 | .77 |  |  |  |  |  |  |  |  |
| Climate for inclusion 11 | .75 |  |  |  |  |  |  |  |  |
| Climate for inclusion 12 | .66 |  |  |  |  |  |  |  |  |
| Career 1 |  |  |  |  | .82 |  |  |  |  |
| Career 2 |  |  |  | .42 |  |  |  |  |  |
| Career 3 |  |  |  |  | .80 |  |  |  |  |
| Career 4 |  |  |  |  | .82 |  |  |  |  |
| Career 5 |  |  |  | .50 | .33 |  |  |  |  |
| Career 6 |  |  |  |  | .51 |  |  |  |  |
| Motivation 1 |  |  |  | .71 |  |  |  |  |  |
| Motivation 2 |  |  |  | .88 |  |  |  |  |  |
| Motivation 3 |  |  |  | .88 |  |  |  |  |  |
| Motivation 4 |  |  |  | .40 |  |  |  | .34 |  |
| Motivation 5 |  |  |  | .78 |  |  |  |  |  |
| Job Satisfaction 1 |  |  |  |  |  |  |  | .80 |  |
| Job Satisfaction 2 |  |  |  |  |  |  |  | .88 |  |
| Job Satisfaction 3 |  |  |  |  |  |  |  | .82 |  |
| Stress 1 |  |  | .81 |  |  |  |  |  |  |
| Stress 2 |  |  | .81 |  |  |  |  |  |  |
| Stress 3 |  |  | .79 |  |  |  |  |  |  |
| Stress 4 |  |  | .82 |  |  |  |  |  |  |
| Stress 5 |  |  | .82 |  |  |  |  |  |  |
| Stress 6 |  |  | .76 |  |  |  |  |  |  |
| Turnover 1 |  |  |  |  |  |  |  |  | .74 |
| Turnover 2 |  |  |  |  |  |  |  |  | .83 |
| Turnover 3 |  |  |  |  |  |  |  | .35 | .41 |
| Turnover 4 |  |  |  |  |  |  |  |  | .47 |
| Eigenvalue | 8.12 | 6.38 | 4.08 | 3.70 | 3.86 | 3.44 | 2.75 | 2.87 | 1.96 |

*Table F.* Descriptives of Inclusion, Job Satisfaction, Work-related Stress, Turnover Intentions, Career Commitment and Career advancement motivation per Dissimilarity Type

|  | Deep-level and Surface-level Similarity | | | Only Deep-level Dissimilarity | | | Only Surface-level Dissimilarity | | | Deep-level and Surface-level Dissimilarity | | |
| --- | --- | --- | --- | --- | --- | --- | --- | --- | --- | --- | --- | --- |
| Variables | *M* | *SD* | *n* | *M* | *SD* | *n* | *M* | *SD* | *n* | *M* | *SD* | *n* |
| Felt Inclusion | 5.43 | 0.95 | 551 | 4.89 | 1.05 | 147 | 5.37 | 0.99 | 111 | 4.62 | 1.74 | 67 |
| Job Satisfaction | 5.75 | 1.00 | 550 | 5.41 | 1.17 | 147 | 5.88 | 0.91 | 111 | 5.34 | 1.26 | 67 |
| Work-related Stress | 4.07 | 0.89 | 550 | 3.89 | 0.90 | 147 | 4.14 | 0.97 | 111 | 3.61 | 1.23 | 67 |
| Turnover Intentions | 0.66 | 0.33 | 551 | 0.57 | 0.36 | 147 | 0.67 | 0.34 | 111 | 0.58 | 0.35 | 67 |
| Career Commitment | 4.83 | 1.02 | 551 | 4.64 | 1.02 | 147 | 5.10 | 1.07 | 111 | 5.02 | 1.34 | 67 |
| Career advancement motivation | 4.44 | 0.85 | 551 | 4.36 | 0.85 | 147 | 4.63 | 0.95 | 111 | 4.55 | 0.90 | 67 |

| *Table G.* Unstandardized Regression Coefficients, Standard Errors, and Model Summary for Two Moderated Mediation Models with Job Satisfaction and Work-related Stress as Dependent Variables using PROCESS with 5000 Bootstraps | | | | | | | | | | | | | | | | | | | | | | | | | | | | | | | | | | | |  |  |
| --- | --- | --- | --- | --- | --- | --- | --- | --- | --- | --- | --- | --- | --- | --- | --- | --- | --- | --- | --- | --- | --- | --- | --- | --- | --- | --- | --- | --- | --- | --- | --- | --- | --- | --- | --- | --- | --- |
| Consequent | | | | | | | | | | | | | | | | | | | | | | | | | | | | | | | | | | | |  |  |
|  | | Felt Inclusion*  *M* | | | | | | | |  | | Job Satisfaction   *Y_1_* | | | | | | | | | | | | Work-related Stress  *Y_2_* | | | | | | | | | |  |  |  |  |
| Antecedent | | *Coeff.* | | *SE* | | *p* | | *95% CI* | |  | |  | | *Coeff.* | | | | | *SE* | | *p* | *95% CI* | | | | | | *Coeff.* | *SE* | | *p* | | *95% CI* | | | | |
| *X_1_* (Deep-level Dissimilarity) |  | | -0.25 | | 0.07 | | < .001 | | -0.400, -0.129 | |  | | | | -0.07 | | | 0.07 | | .318 | | | -0.214, 0.070 | | |  | -0.08 | | 0.07 | .279 | | -0.218, 0.063 | | | | |  |
| *X_2_* (Surface-level Dissimilarity) |  | | -0.01 | | 0.08 | | .898 | | -0.148, 0.185 | |  | | | | 0.13 | | | 0.08 | | .079 | | | -0.015, 0.279 | | |  | -0.01 | | 0.07 | .943 | | -0.151, 0.141 | | | | |  |
| *W* (Climate for Inclusion) |  | | 0.55 | | 0.03 | | < .001 | | 0.484, 0.617 | |  | | | |  | | |  | |  | | |  | | |  |  | |  |  | |  | | | | |  |
| *M* (Felt Inclusion) |  | |  | |  | |  | |  | |  | | | | 0.53 | | | 0.03 | | < .001 | | | 0.474, 0.585 | | |  | 0.31 | | 0.03 | < .001 | | 0.258,  0.369 | | | | |  |
| *X_1_* x *W* |  | | 0.27 | | 0.06 | | < .001 | | 0.145, 0.394 | |  | | | |  | | |  | |  | | |  | | |  |  | |  |  | |  | | | | |  |
|  |  | |  | |  | |  | |  | |  | |  | | |  |  | | |  | | |  | |  |  | | |  | | |  | | |  |  |  |
| Conditional Indirect effect of *X_1_* on *Y*, at mean value of *W* |  | |  | |  | |  | |  | |  | |  | | | -0.13 | 0.04 | | |  | | | -0.217, -0.059 | |  | -0.08 | | | 0.02 |  | | -0.132, -0.036 | | |  |  |  |
|  |  | |  | |  | | *R*² = 0.35 | |  | |  | |  | | |  | *R*² = 0.30 | | |  | | |  | |  |  | | | *R*² = 0.14 | | |  | | |  |  |  |

* The results for felt inclusion as consequent differed slightly in the analyses, in that only the confidence intervals differed. For simplicity, we presented the confidence interval in the PROCESS model with job satisfaction as the dependent variable.

| *Table H.* Unstandardized Regression Coefficients, Standard Errors, and Model Summary for Three Moderated Mediation Models with Turnover Intentions, Career Commitment and Career Advancement Motivation as Dependent Variables using PROCESS with 5000 Bootstraps | | | | | | | | | | | | | | | | | | |
| --- | --- | --- | --- | --- | --- | --- | --- | --- | --- | --- | --- | --- | --- | --- | --- | --- | --- | --- |
| Consequent | | | | | | | | | | | | | | | | | | |
|  | | *Y_3_* (Turnover Intentions) | | | | | *Y_4_* (Career Commitment) | | | | |  | *Y_5_* (Career Advancement Motivation) | | | | | |
| Antecedent | | *Coeff.* | *SE* | *p* | *95% CI* | | *Coeff.* | *SE* | *p* | *95% CI* |  | *Coeff.* | *SE* | | *p* | | *95% CI* | |
| *X_1_* (Deep-level Dissimilar) |  | -0.05 | 0.03 | .092 | -0.100, 0.008 |  | -0.14 | 0.09 | .134 | -0.314, 0.042 |  | 0.08 | 0.07 | | .281 | | -0.065, 0.223 | |
| *X_2_* (Surface-level Dissimilar) |  | 0.02 | 0.03 | .490 | -0.036, 0.075 |  | 0.37 | 0.09 | < .001 | 0.183, 0.554 |  | 0.25 | 0.07 | | .001 | | 0.105, 0.404 | |
| *M* (Felt Inclusion) |  | 0.07 | 0.01 | < .001 | 0.046,  0.089 |  | 0.10 | 0.04 | .003 | 0.034, 0.174 |  | 0.24 | 0.03 | | < .001 | | 0.186, 0.299 | |
| Conditional Indirect effect of *X_1_* on *Y*, at mean value of *W* |  | -0.02 | 0.01 |  | -0.030, -0.008 |  | -0.03 | 0.01 |  | -0.063, -0.005 |  | -0.06 | 0.02 | |  | | -0.104, -0.027 | |
|  |  |  | *R*² = 0.05 |  |  | |  |  | *R*² = 0.03 |  |  |  |  | *R*² = 0.08 | |  | |  |

| *Table I.* Model Coefficients for the Conditional Model on Inclusion using SEM | | | | | | |
| --- | --- | --- | --- | --- | --- | --- |
| *M* (Felt Inlusion) | | | | | | |
| Variables | Level of Climate for Inclusion *W* | | *Coeff.* | *SE* | *p* | *95% CI* |
| *X_1_* (Deep-level Dissimilarity) | | -1 SD | -0.45 | 0.11 | < .001 | -0.652, -0.241 |
|  | | Mean | -0.27 | 0.08 | < .001 | -0.411, -0.129 |
|  | | +1 SD | -0.09 | 0.10 | .369 | -0.297, 0.110 |
| *X_2_* (Surface-level Dissimilarity) | | -1 SD | -0.11 | 0.15 | 0.478 | -0.405, 0.109 |
|  | | Mean | 0.02 | 0.09 | 0.827 | -0.148, 0.185 |
|  | | +1 SD | 0.15 | 0.10 | 0.152 | -0.053, 0.343 |

| *Table J*. Regression Coefficients, Standard Errors, and Model Summary for Felt Inclusion and Work-related Outcomes in the Moderated Mediation Model with Latent Variable ‘Work-related Outcomes’ | | | | | | | | | | | | | | | | |
| --- | --- | --- | --- | --- | --- | --- | --- | --- | --- | --- | --- | --- | --- | --- | --- | --- |
|  | | Felt Inclusion (*M*) | | | | | | | Work-related Outcomes (*Y_1_*) | | | | | | | |
| Antecedent | | *Coeff.* | | *SE* | *p* | *95% CI* | |  | *Coeff.* | | *SE* | *p* | *95% CI* | | |  |
| *X_1_* (Deep-level Dissimilarity) | *a_1_* | -0.27 | | 0.07 | < .001 | -0.411, -0.129 | | c’_1_ | -0.10 | 0.11 | | .327 | | -0.326, 0.109 | |  |
| *X_2_* (Surface-level Dissimilarity) | *a_2_* | 0.02 | | 0.09 | .826 | -0.148, 0.185 | | c’_2_ | 0.21 | 0.11 | | .052 | | | -0.002, 0.412 |  |
| *W* (Climate for Inclusion) | *a_3_* | 0.47 | | 0.03 | < .001 | 0.407, 0.539 | |  |  |  | |  | | |  |  |
| *M* (Felt Inclusion) |  |  | |  |  |  | | *b_1_* | 0.83 | 0.07 | | < .001 | | | 0.681, 0.972 |  |
| *X_1_* x *W* | *a_4_* | 0.18 | | 0.08 | .019 | 0.029, 0.328 | |  |  |  | |  | | |  |  |
| *X_2_* x *W* | *a_5_* | 0.13 | | 0.10 | .192 | -0.064, 0.319 | |  |  |  | |  | | |  |  |
| *X_1_* x *X_2_* | *a_6_* | 0.03 | | 0.19 | 0.883 | -0.339, 0.395 | |  |  |  | |  | | |  |  |
| *X_1_* x *X_2_ x W* | *a_7_* | 0.06 | | 0.15 | 0.692 | -0.236, 0.356 | |  |  |  | |  | | |  |  |
|  |  | |  | *R²* = .27 |  |  |  | |  | *R²* = .42 | |  | | |  |  |

*Table K.* Descriptives of Authenticity and Belonging per Dissimilarity Type

|  | Deep-level and Surface-level Similarity | | | Only Deep-level Dissimilarity | | | Only Surface-level Dissimilarity | | | Deep-level and Surface-level Dissimilarity | | |
| --- | --- | --- | --- | --- | --- | --- | --- | --- | --- | --- | --- | --- |
| Variables | *M* | *SD* | *n* | *M* | *SD* | *n* | *M* | *SD* | *n* | *M* | *SD* | *n* |
| Authenticity | 5.24 | 1.05 | 551 | 4.64 | 1.16 | 147 | 5.26 | 1.05 | 111 | 4.38 | 1.87 | 67 |
| Belonging | 5.62 | 0.97 | 551 | 5.14 | 1.07 | 147 | 5.47 | 1.04 | 111 | 4.86 | 1.78 | 67 |

1. This analysis was repeated after removal of outliers (+- 3 SD), yielding similar results. [↑](#footnote-ref-1)
2. Per the request of the editor [↑](#footnote-ref-2)
3. We did include surface-level dissimilarity as a covariate in the analyses. The results were mostly the same, except that surface-level dissimilarity predict career commitment and career advancement motivation as described in section 1.3 above. [↑](#footnote-ref-3)
